# The Effect of Probiotics on the Improvement of Body Weight and Fat

**DOI:** 10.1002/fsn3.70728

**Published:** 2025-08-06

**Authors:** Hongfa Zhang, Yu Xu, Mingjie Li, Zhaowei Chen, Yuezhu Wang, Rongfu Yang, Sheng Li, Shiyu Ma, Chunping You, Huajun Zheng

**Affiliations:** ^1^ State Key Laboratory of Dairy Biotechnology, Shanghai Engineering Research Center of Dairy Biotechnology Dairy Research Institute, Bright Dairy & Food Co., Ltd., Synergetic Innovation Center of Food Safety and Nutrition Shanghai China; ^2^ Shanghai‐MOST Key Laboratory of Health and Disease Genomics, NHC Key Lab of Reproduction Regulation Shanghai Institute for Biomedical and Pharmaceutical Technologies Shanghai China

**Keywords:** body weight, gut microbiota, *Lactiplantibacillus plantarum*, obesity

## Abstract

This study aimed to investigate the effects of *Lactiplantibacillus plantarum* ( *L. plantarum* ) ST‐Ⅲ and oats supplementation on high‐fat diet (HFD)–induced obesity in rats, focusing on changes in body weight, organ/fat tissue weights, inflammatory markers, serum parameters, and gut microbiota composition. Male Sprague–Dawley rats (*n* ≥ 6/group) were fed a normal diet (ND) or HFD for 9 weeks to establish obesity. Then, HFD‐fed rats were divided into four groups: HFD + ST‐Ⅲ, ND + ST‐Ⅲ, HFD + Oats, and ND + Oats, receiving daily oral gavage of ST‐III (1.5 × 10^9^ CFU/rat) or oats for another 9 weeks. Body weight, serum lipids, cytokines (e.g., IL‐6, PYY, and MCP‐1), liver histopathology, and gut microbiota were analyzed. Notably, weight loss is primarily influenced by diet, and better results can be achieved by controlling diet in combination with new edible *L. plantarum* ST‐Ⅲ in promoting weight loss. *L. plantarum* ST‐Ⅲ reduced the levels of the factors CORT and MCP‐1, thereby potentially promoting weight loss. The ST‐Ⅲ group showed earlier weight reduction (significant at Week 2) than the oats group (at Week 6). Gut microbiota analysis revealed differential abundances of *Lactiplantibacillus*, *Blautia*, and *Prevotella* between intervention groups. No significant differences in liver or organ weights were observed. *L. plantarum* ST‐Ⅲ and oats attenuated HFD‐induced obesity by modulating metabolic parameters, inflammation, and gut microbiota. ST‐Ⅲ exhibited quicker anti‐obesity effects, while oats enhanced probiotic growth. Dietary structure improvement remains critical for weight management. Further studies should address stress‐related experimental limitations and explore clinical applications.

## Graphical abstract

Weight loss is primarily influenced by diet, and better results can be achieved by controlling diet in combination with new edible *L. plantarum* ST‐Ⅲ in promoting weight loss. The *L. plantarum* ST‐Ⅲ used in this study may have beneficial anti‐obesity effects.

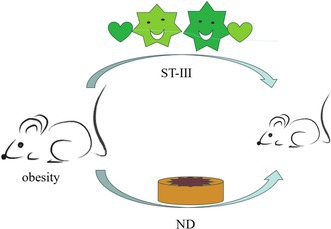

## 1 Introduction

Obesity is a key feature of several metabolic and chronic ailments and increases the risk of diseases, especially type 2 diabetes mellitus, hyperlipidemia, and cardiovascular diseases (Hsu et al. 2021). Obesity stems from an imbalance between the amount of energy consumed and the amount of energy expended. This is due mainly to a high‐fat diet (HFD) and lack of physical exercise (2022). Recent studies have focused on HFD‐induced gut microbiota dysbiosis, dyslipidemia, inflammation, and nonalcoholic fatty liver disease (NAFLD) (Zhou et al. 2022).

Currently, pharmacological interventions do not seem to be efficacious in achieving enduring long‐term weight reduction (Twardowska et al. 2022). Physical exercise and healthy eating have been widely demonstrated to be effective therapies for preventing obesity (Hsu et al. 2021) and improving metabolic function in HFD‐induced obese rats. The use of probiotics has been suggested as a promising therapeutic strategy for treating obesity and related disorders (Zhou et al. 2022).

Previous researchers have shown that probiotics can improve metabolic health (Liu, Zheng, et al. 2022), ameliorate hyperlipidemia and inflammation (Freire et al. 2021; Chen et al. 2022), and alleviate NAFLD (Ding et al. 2022) in HFD‐fed rats. Probiotics such as *L. plantarum* dfa1 alleviate obesity in HFD‐induced obese mice (Ondee et al. 2022). *L. plantarum* LMT1‐48 exerts an anti‐obesity effect through the PPAR‐alpha signaling pathway (Zhu et al. 2019) and regulates adipogenesis (Rahman et al. 2021). Epididymal and renal adipose tissue weights are significantly lower in HFD‐fed mice fed heat‐killed *Lactobacillus brevis* KB290 (Watanabe et al. 2021). Other studies have confirmed that *L. plantarum* PH04 and *L. plantarum* LP104 attenuate serum cholesterol and triglyceride levels in hypercholesterolemic mice (Nguyen et al. 2006). Probiotics reduce oxidative stress and regulate immune responses in a rat model (Liu, Zhao, et al. 2022). *L. plantarum* N‐1 enhances intestinal barrier function (Wei et al. 2021). *Lactobacillus casei* improves adverse intestinal reactions in rats by modulating the gut microbiota and short‐chain fatty acids (Li et al. 2022).

Oats are healthy foods that reduce body weight, blood serum cholesterol levels, the inflammatory state, and glucose metabolism (Peipei et al. 2010). Oats possess potent antioxidant properties and exhibit high antioxidative activity. Consequently, they are increasingly recognized as functional foods with significant probiotic potential (Alemayehu et al. 2023). The prebiotic capability of oats improved the growth of the probiotic strains (Kalpa et al. 2021). Previous researchers have shown that the combination of inulin and a compound probiotic synergistically attenuates HFD‐induced obesity (Wang et al. 2020). Compared with the addition of free prebiotic, the addition of oats enhances the storage stability and survival of probiotics (Chen et al. 2020; Kalpa et al. 2021). The study revealed that the combination of oats and *L. plantarum* has potential antidiabetic effects on rats (Alharbi et al. 2022). Moreover, the weight loss effect of oats combined with a normal diet (ND) on overweight is much greater than that of a ND alone (Ding et al. 2017).

Therefore, the purpose of this study was to investigate the effects of dietary structure or *L. plantarum* ST‐Ⅲ on HFD‐induced obesity in rats, including changes in body, organ, and fat tissue weights; inflammatory cytokine signatures; serum parameters; and the gut microbiota.

## 2 Materials and Methods

### 2.1 Fermented Oats and Probiotics

The oats and *L. plantarum* ST‐Ⅲ used in this study were in broth form and provided by Brightdairy Co. Ltd. (China). The dry oat flour (3%), fresh and homogeneous pineapple pulp (10%), and water (87%) were mixed evenly and incubated at 65°C for 4 h. By hydrolyzing the proteins in oats with bromelain, the necessary substances for the growth of *L. plantarum* ST‐Ⅲ are provided (Hongfa et al. 2024). After the samples were filtered through two layers of gauze, they were sterilized at 115°C for 10 min to prepare oat beverages (Oats). *L. plantarum* ST‐Ⅲ at a final concentration of approximately 1 × 10^7^ CFU/mL was fermented at 37°C for 16 h and cooled to 4°C for later use. The fermentation sample of *L. plantarum* ST‐Ⅲ was diluted with water in a 10‐fold gradient. Suitable dilutions were selected, and 100 μL aliquots were spread on MRS agar plates. The plates were incubated anaerobically at 37°C for 72 h, and the colonies were counted. The suspension of *L. plantarum* ST‐Ⅲ was adjusted to a final concentration of 5 × 10^8^ CFU/mL for used (ST‐Ⅲ).

### 2.2 Animals and Treatment

Male Sprague–Dawley (SD) rats (aged 4 weeks, *n* ≥ 6 per group) were obtained from Shanghai JieSiJie Laboratory Animal Co. Ltd. (China). The rats were housed under a 12 h light/dark cycle under controlled conditions with a room temperature of 23°C ± 1°C and a humidity of 50% ± 10%.

**FIGURE 1:**
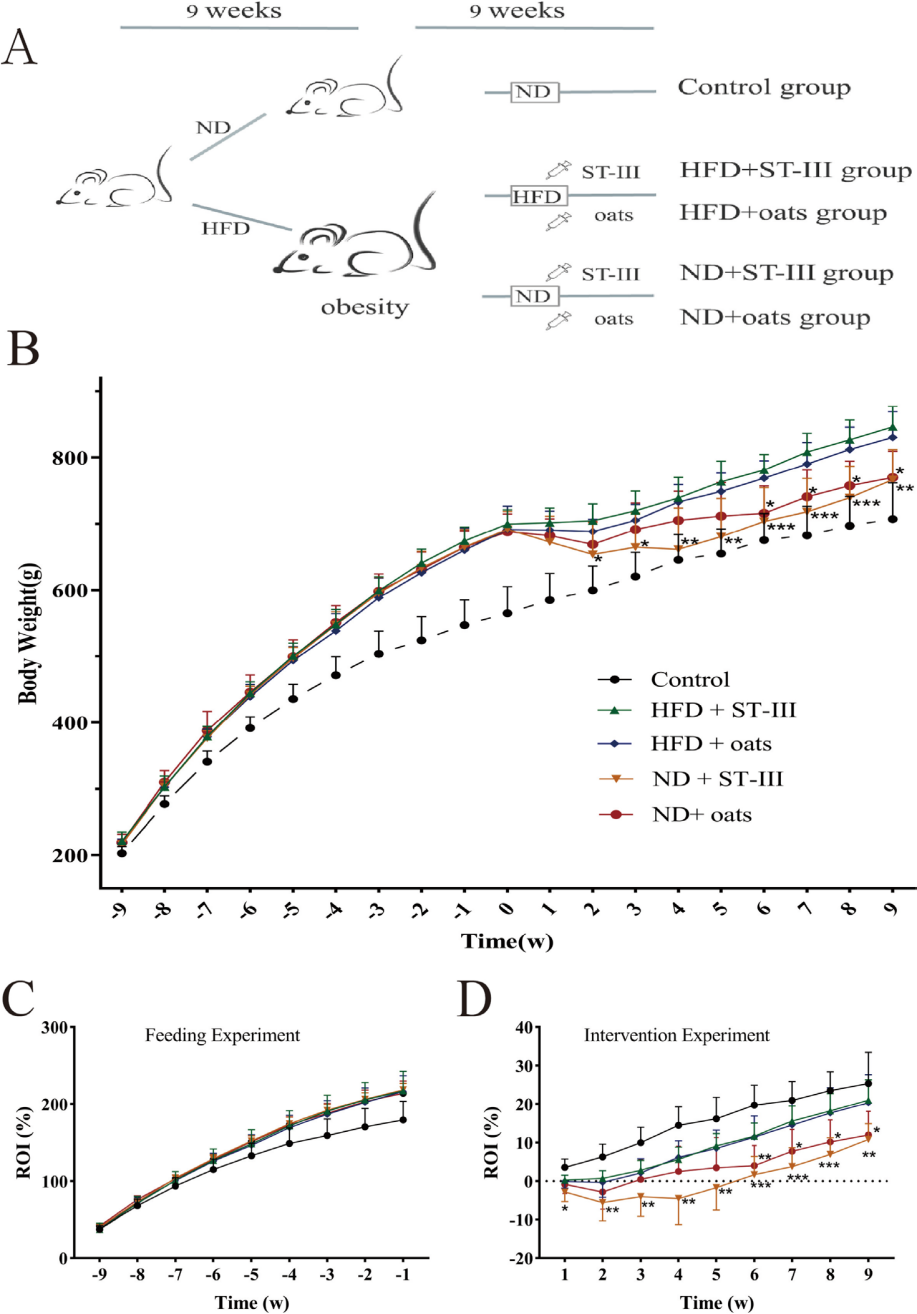
Effects of the probiotic and oats interventions on growth. Obesity was induced by a HFD for 9 weeks, and this was followed by the interventions over 9 weeks. (A) Schematic of ST‐Ⅲ and oats on obesity model. The animals were randomly assigned to the indicated five groups (Control, HFD + ST‐Ⅲ, HFD + oats, ND + ST‐Ⅲ, and ND + oats group). (B) Weekly body weight changes of Sprague–Dawley (SD) rats. (C) Weekly body rate of increase (ROI) changes of SD rats in feeding experiment. There was no significant difference in weight gain rate among rats with successful obesity modeling. (D) Weekly body ROI changes of SD rats in intervention experiment. In rats with successful obesity modeling, there was no significant functional difference between ST‐Ⅲ and oats under high‐fat diet conditions (comparing Group HFD + ST‐Ⅲ with Group HFD + oats). However, under normal diet conditions (comparing Group ND + ST‐Ⅲ with Group ND + oats), ST‐Ⅲ had a better weight loss effect than oats (*p >* 0.05). The asterisk labeled on ND+ST‐Ⅲ group indicated significant changes compared with HFD+ST‐Ⅲ group and the asterisk labeled on ND+oats group indicated significant changes compared with HFD+oats group (*n* ≥ 6, * *p* < 0.05; **p < 0.01; *** *p* < 0.001).

The study was designed with two phases of experiments, which took a total duration of 18 weeks. Phase 1 is the process of obesity model construction. Following a 2‐week acclimatization period on a normal diet, the rats were randomly assigned to be fed a ND (control, *n* = 9) (13.8% kcal from fat, 1,010,083; Jiangsu Xietong Pharmaceutical Bio‐Engineering Co. Ltd., China) or a HFD (obese group, *n* = 29) (37% kcal from fat, sucrose was replaced with fructose, TP0860; Trophic Animal Feed High‐Tech Co. Ltd., China) for 9 weeks. As shown in Table 1, the Phase 2 experiment involved probiotic or oat intervention for another 9 weeks. The control group continued to be fed a ND, and the obesity group was divided into four treatment groups (Figure 1A): HFD + ST‐Ⅲ (HFD supplemented with *L. plantarum* ST‐Ⅲ), ND + ST‐Ⅲ (ND supplemented with *L. plantarum* ST‐Ⅲ), HFD + Oats (HFD supplemented with oats), and ND + Oats (ND supplemented with oats). ST‐Ⅲ or oats were given 3 mL/rat/day from 10:00–11:00 through gavage. The daily dose of ST‐III was 1.5 × 10^9^ CFU/rat. All the rats were allowed free access to food and water. The body weight was determined weekly.

**TABLE 1:** Intervention groups in this study.

Groups	Rats (*n*)	Descriptions	Treatments^a^
Control	9	Control rats	/
HFD + ST‐Ⅲ	6	Obese rats	3 mL of ST‐Ⅲ (1.5 × 10^9^ CFU/rat)
ND + ST‐Ⅲ	8	Obese rats	3 mL of ST‐Ⅲ (1.5 × 10^9^ CFU/rat)
HFD + oats	8	Obese rats	3 mL of oats
ND + oats	7	Obese rats	3 mL of oats

Abbreviations: Control, normal diet in two phases; HFD, high‐fat diet; ND, normal diet; ST‐Ⅲ, *Lactobacillus plantarum* ST‐Ⅲ.

^a^ Treatments were given orally once a day for 9 weeks during the Phase 2 experiment.

At the end of the experimental period, the rats were fasted for 12 h. The rats were sacrificed by withdrawing blood from the abdominal aorta under 10% chloral hydrate anesthesia. Serum was obtained by centrifuging the blood at 1500 × g for 15 min at 4°C. Furthermore, organs such as the heart, spleen, epididymal fat, perirenal fat, kidney, and liver were removed and weighed. The livers were dissected and immediately immersed in liquid nitrogen. The sera and tissues were stored at −80°C until analysis.

This study was approved by the Ethics Committee on Laboratory Animals of the Shanghai Institute of Planned Parenthood Research (protocol code 2020‐34, approval date 8/20/2020).

### 2.3 Serum Analysis

Triglyceride (TG), total cholesterol (TC), high‐density lipoprotein cholesterol (HDL‐C), and low‐density lipoprotein cholesterol (LDL‐C) levels were analyzed via commercial kits (Nanjing Jiancheng Bioengineering Institute Co., Nanjing, China).

The serum cytokine levels of interleukin (IL)‐6, IL‐1β, growth hormone (GH), leptin (LEP), insulin (INS), glucagons‐like peptide 1 (GLP‐1), cortisol, endotoxin (ET), monocyte chemotactic protein 1 (MCP‐1), and peptide YY (PYY) were determined via ELISA kits (Shanghai Ji Ning Industrial Co. Ltd., Shanghai, China).

### 2.4 Histopathological Analysis

Liver oil red O staining solution was used to prepare the liver, which was filtered, stained, and observed under a microscope within 2 h. The small intestine (duodenum) and large intestine (colon) were excised aseptically. The luminal contents were flushed. The samples were fixed in 10% buffered neutral formalin solution and then dehydrated in graded (70%–100%) alcohol. After being cleared in xylene, each sample was embedded in paraffin. For analysis, the samples were stained with hematoxylin and eosin (H&E) dye and subsequently examined under a light microscope.

### 2.5 Statistical Analysis

The data from individual experiments were analyzed with SPSS software (version 15.030.0.0 for Windows). The results are presented as the mean ± standard error of the mean (SEM). Differences were examined via one‐way ANOVA followed by Tukey's HSD post hoc test (GraphPad Prism, version 10.4.1) for multiple comparisons. Values of *p* < 0.05 were considered significant.

### 2.6 Genomic DNA Extraction, PCR Amplification, and 16S rRNA Gene Sequencing

Fecal samples were collected at the end of the study. DNA was extracted from these samples via the QIAamp DNA Stool Mini Kit from QIAGEN. The 16S rRNA genes were amplified with the primers 338F and 806R, which target the V3–V4 region (Huse et al. 2007), and TransStart Fastpfu DNA Polymerase from TransGen was utilized for this process. The PCR products of each sample were purified with an AxyPrep DNA Gewendul Extraction Kit from Axygen. The purity and concentration of the amplicons were determined via spectrophotometry with the QuantiFluor‐ST system from Promega. The pooled 16S rRNA PCR products were subsequently sequenced via a 2 × 300 bp paired‐end approach on an Illumina MiSeq sequencing platform.

### 2.7 Bioinformatics and Statistical Analysis

The plugin “DADA2” (Benjamin et al. 2016) of QIIME2 (Bolyen et al. 2019) was used to construct an amplicon sequence variant (ASV). Taxonomic assignments post‐normalization were performed via the Ribosomal Database Project (Benjamin et al. 2016) with an 80% threshold. Group differences were evaluated with permutational multivariate analysis of variance (PERMANOVA). PICRUSt2 predicts microbiome functions on the basis of normalized 16S rRNA copy numbers (Douglas et al. 2020). LEfSe was used to analyze taxonomic (ASV, genus, family, and phylum) and functional differences between groups (Segata et al. 2011). Spearman correlation in R was used to calculate species correlations, setting parameters at a coefficient > 0.35 or < −0.35 and *p* < 0.05.

## 3 Results

### 3.1 Changes in Body Weight

The body weights of the rats in all the groups increased weekly (Figure 1B). During Phase 1, the HFD‐fed groups presented a significant increase in body weight compared with the control group (Figure 1C).

In Phase 2, the data demonstrated weight loss in all four intervention groups in the first 2 weeks (Figure 1B,D), and the two HFD groups presented similar trends in terms of body weight increase over the remaining 7 weeks. The body weights of the two ND groups increased every week, especially those of the oat groups, compared with those of the ST‐Ⅲ groups over the 7‐week period. The oats promoted a significant increase in body weight over the next 3 weeks, and the body weights of the two ND groups were similar in the last 4 weeks.

The obese rats that were fed a ND presented lower body weights than the rats fed a HFD did (Figure 1B–D). The differences in body weight between the HFD + ST‐Ⅲ group and the ND + ST‐Ⅲ group became significant (*p <* 0.05) after the second week. The differences in body weight between the HFD + oats group and the ND + oats group became significant (*p* < 0.05) after the sixth week.

These data demonstrated no significant difference in body weight between the HFD + ST‐Ⅲ group and the HFD + oats group. Similarly, there was no significant difference between the ND + ST‐Ⅲ group and the ND + oats group.

### 3.2 HFD‐Induced Obesity Increases Liver, Small Intestine, and Large Intestine Tissue Damage

**FIGURE 2:**
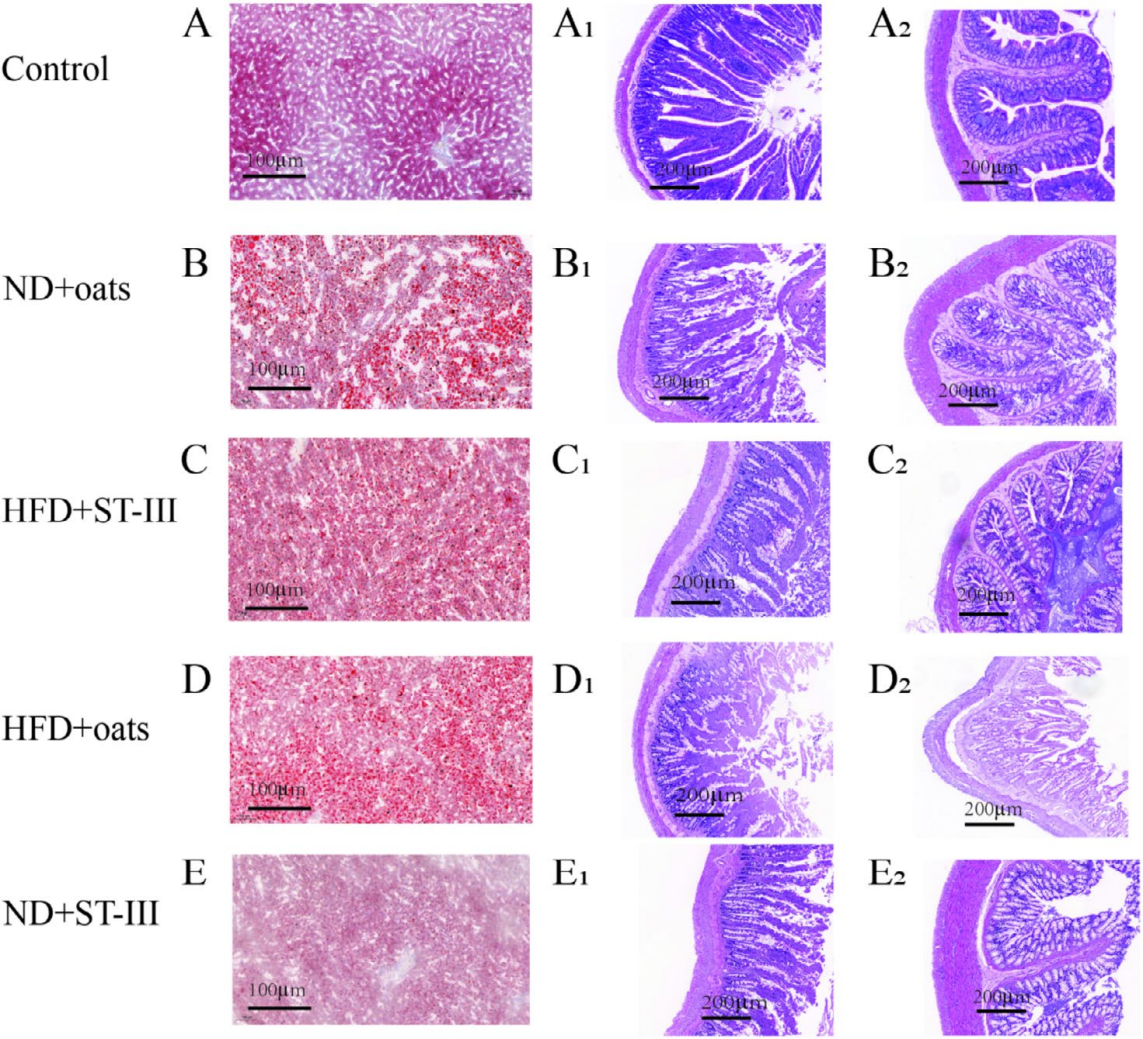
Histopathological changes in liver and intestinal tissues. Liver sections were stained with Oil Red O to visualize lipid droplets (first column: A‐E). Duodenum (second column: A1‐E1) and colon (third column: A2‐E2) sections were stained with hematoxylin and eosin (H&E) to assess histology. Images were captured using light microscopy at 5× magnification. Panels display representative sections from each experimental group.

To assess the effects of the HFD on morphological changes, the liver weight was measured. As shown in Table 2, there was no significant difference in tissue weight. Lipid droplets in the liver were visualized via oil (red) O staining. In the ND group, small and evenly distributed red lipid droplets were visible in the control group (Figure 2A). However, liver cells that were swollen had red lipid droplets in the ND + oats group (Figure 2B).

**TABLE 2:** Tissue weights at the end of study.

Weight (g)	Control	HFD + ST‐Ⅲ	HFD + oats	ND + ST‐Ⅲ	ND + oats
	Mean ± SD	Mean ± SD	Mean ± SD	Mean ± SD	Mean ± SD
Heart	2.16 ± 0.22	2.20 ± 0.28	2.17 ± 0.29	2.30 ± 0.46	2.41 ± 0.30
Spleen	0.96 ± 0.20	1.00 ± 0.13	1.26 ± 0.32	1.15 ± 0.43	1.11 ± 0.18
Liver	21.60 ± 2.66	18.23 ± 6.06	22.75 ± 1.80	22.63 ± 3.59	21.26 ± 1.90
Kidney	3.78 ± 0.86	4.31 ± 0.38	5.42 ± 1.86	5.34 ± 0.81	6.16 ± 2.04
Perirenal fat	16.19 ± 5.83	32.79 ± 22.68	25.21 ± 14.62	25.27 ± 13.44	22.50 ± 11.87
Epididymal fat	12.66 ± 3.04	25.91 ± 3.39**	26.46 ± 9.28*	14.63 ± 1.62	17.57 ± 2.29

*Note:* Each value is expressed as mean ± SD. Mean values within a row with different superscript differ significantly (HFD + ST‐Ⅲ vs. ND + ST‐Ⅲ, HFD + oats vs. ND + oats group). *n* ≥ 6.

* *p* < 0.05.

** *p* < 0.01.

As shown in Figure 2, compared with the control group, the ND + oats group presented with intestinal lesions and destruction of the epithelium in the small intestine ileum, as observed through hematoxylin–eosin (H&E) staining (5×). In addition, histological changes revealed a decrease in villus height in the ND + oats group compared with the control group.

### 3.3 Effects on Visceral Organ Weight and Fasting Blood Glucose

**FIGURE 3:**
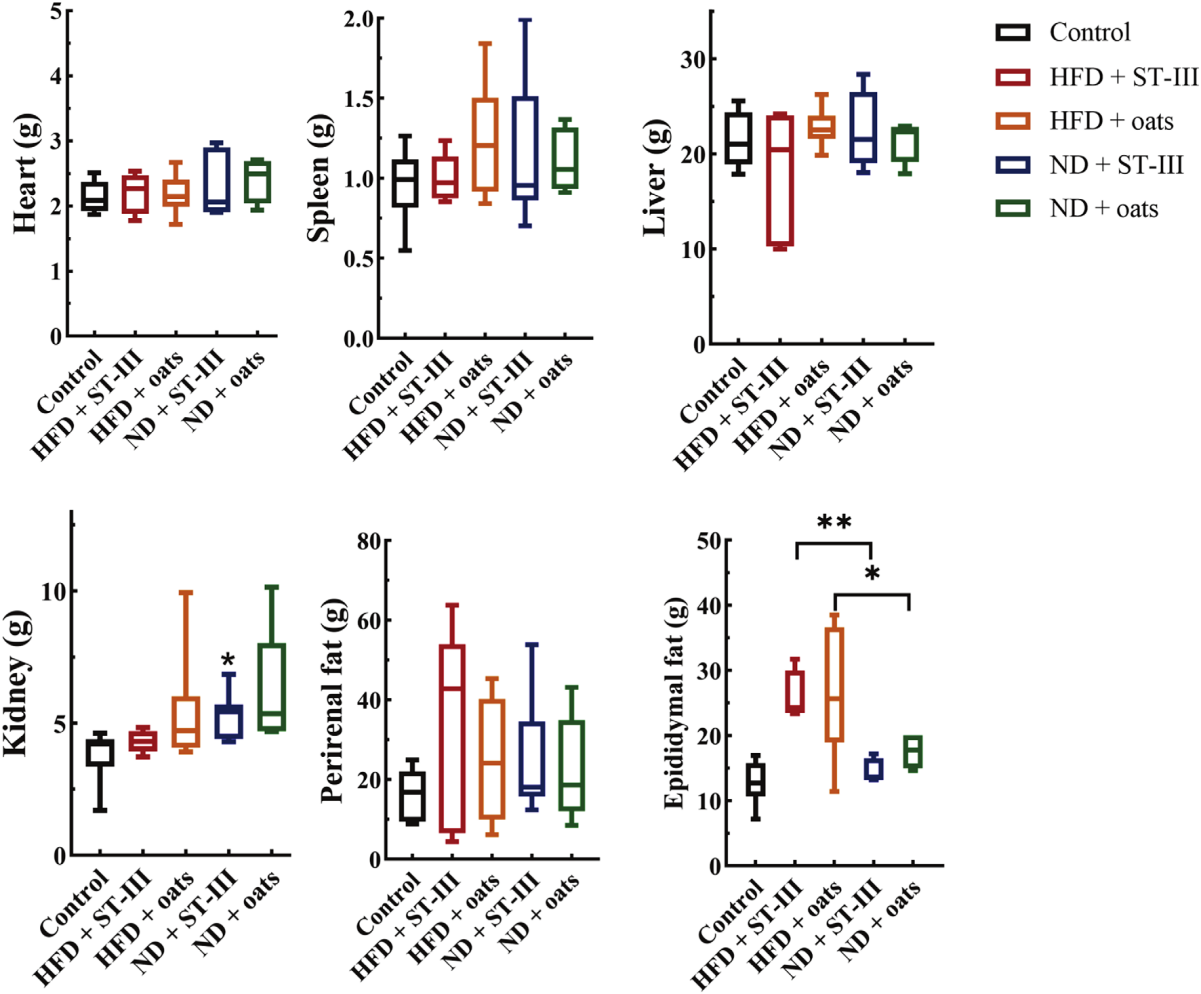
Tissue weights. Comparing group HFD + ST‐Ⅲ with group ND + ST‐Ⅲ (HFD + ST‐Ⅲ vs. ND + ST‐Ⅲ) revealed that diet (normal and high fat) had a significant impact on the epididymal fat of rats (*p* < 0.05). Each value is expressed as mean ± SD. * *p* < 0.05, **p < 0.01, *n* ≥ 6.

As shown in Figure 3, it was observed that there was no significant difference in visceral organ weight between the ST‐Ⅲ and oats groups. Heart, spleen, kidney, and liver weights were compared between the ND group and the HFD group, but no significant differences were detected. However, the rats in the ND group treated with ST‐Ⅲ (ND + ST‐Ⅲ group) presented significantly (*p* < 0.05) lower epididymal fat (E‐fat) weights than those in the HFD + ST‐Ⅲ group.

**FIGURE 4:**
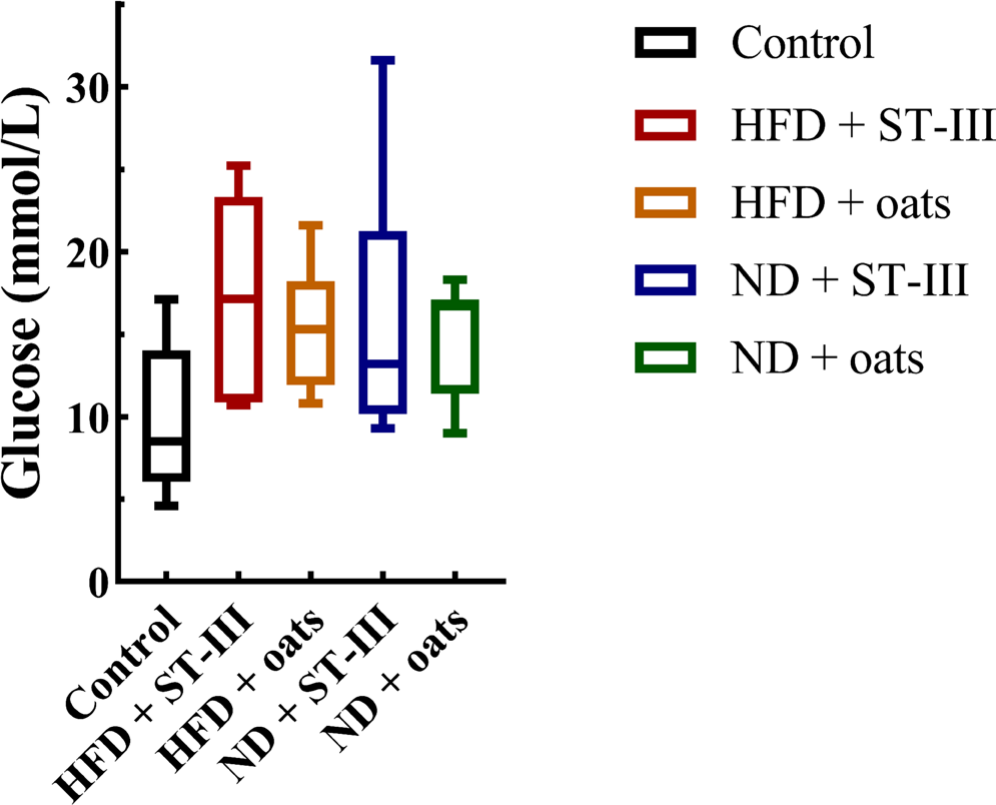
Fasting blood glucose levels at the end of study. Each value is expressed as mean ± SD. No significant difference was observed between groups, *n* ≥ 6.

The obese group presented greater fasting blood glucose (FBG) levels than the control group did (*p* < 0.05) (Figure 4).

Compared with the HFD + ST ‐ Ⅲ group, the ND + ST‐Ⅲ group presented a lower FBG level. Similarly, a lower FBG level was observed in the ND + oats group than that in the HFD + oats group. However, the FBG levels were not significantly different between the HFD and ND groups.

### 3.4 Hypolipidemic and Hypocholesterolemic Effects of the Oral Administration of *L. plantarum* ST‐Ⅲ or Oats in Obese Rats

**FIGURE 5:**
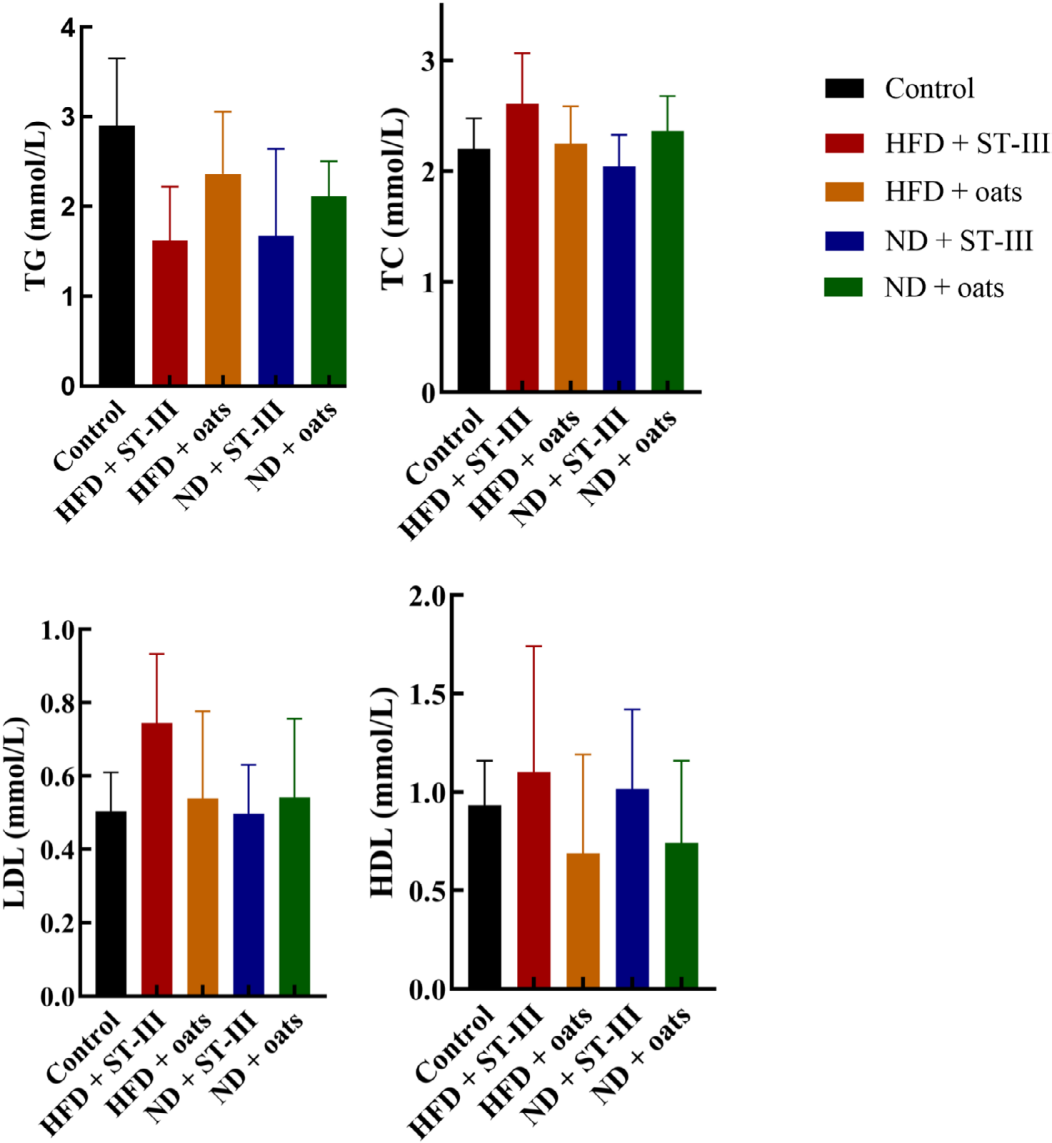
The serum (TG, TC, HDL‐C, and LDL‐C) lipid profiles. Each value is expressed as mean ± SD. Values of each group were not significantly different. *n* ≥ 6. HDL‐C, high‐density lipoprotein cholesterol; LDL‐C, low‐density lipoprotein cholesterol; TC, total cholesterol; TG, triglyceride.

Although the TC, TG, LDL, and HDL levels in the ND group were almost lower than those in the HFD group (Figure 5, Table 3), the differences were not significant (the HFD + ST‐Ⅲ group vs. the ND + ST‐Ⅲ group, the HFD + oats group vs. the ND + oats group). Compared with the oats group, supplementation with ST‐Ⅲ caused decreases in TC, TG, and LDL in the ND group (ND + ST‐Ⅲ group vs. ND + oats group). Compared with those in the ST‐Ⅲ group, the oats in the HFD group presented decreases in TC, LDL, and HDL (groups HFD + ST‐Ⅲ vs. HFD + Oats).

**TABLE 3:** Lipid concentration in serum at the end of study.

Concentration	Control	HFD + ST‐Ⅲ	HFD + oats	ND + ST‐Ⅲ	ND + oats
	Mean ± SD	Mean ± SD	Mean ± SD	Mean ± SD	Mean ± SD
Glucose (mmol/L)	9.88 ± 4.21	17.3 ± 5.43	15.41 ± 3.40	15.93 ± 7.31	14.87 ± 3.18
TG (mmol/L)	2.90 ± 0.71	1.62 ± 0.55	2.37 ± 0.65	1.67 ± 0.91	2.11 ± 0.35
TC (mmol/L)	2.20 ± 0.26	2.61 ± 0.42	2.25 ± 0.31	2.04 ± 0.27	2.36 ± 0.29
HDL‐C (mmol/L)	1.03 ± 0.20	1.52 ± 0.35	1.11 ± 0.45	1.02 ± 0.25	1.11 ± 0.40
LDL‐C (mmol/L)	1.60 ± 0.37	1.89 ± 1.00	1.45 ± 0.75	1.74 ± 0.65	1.27 ± 0.62

*Note:* Each value is expressed as mean ± SD.

Abbreviations: HDL‐C, high‐density lipoprotein cholesterol; LDL‐C, low‐density lipoprotein cholesterol; TC, total cholesterol; TG, triglyceride.

### 3.5 ST‐Ⅲ Intervention Decreases the Levels of Host Inflammatory Factors

**FIGURE 6:**
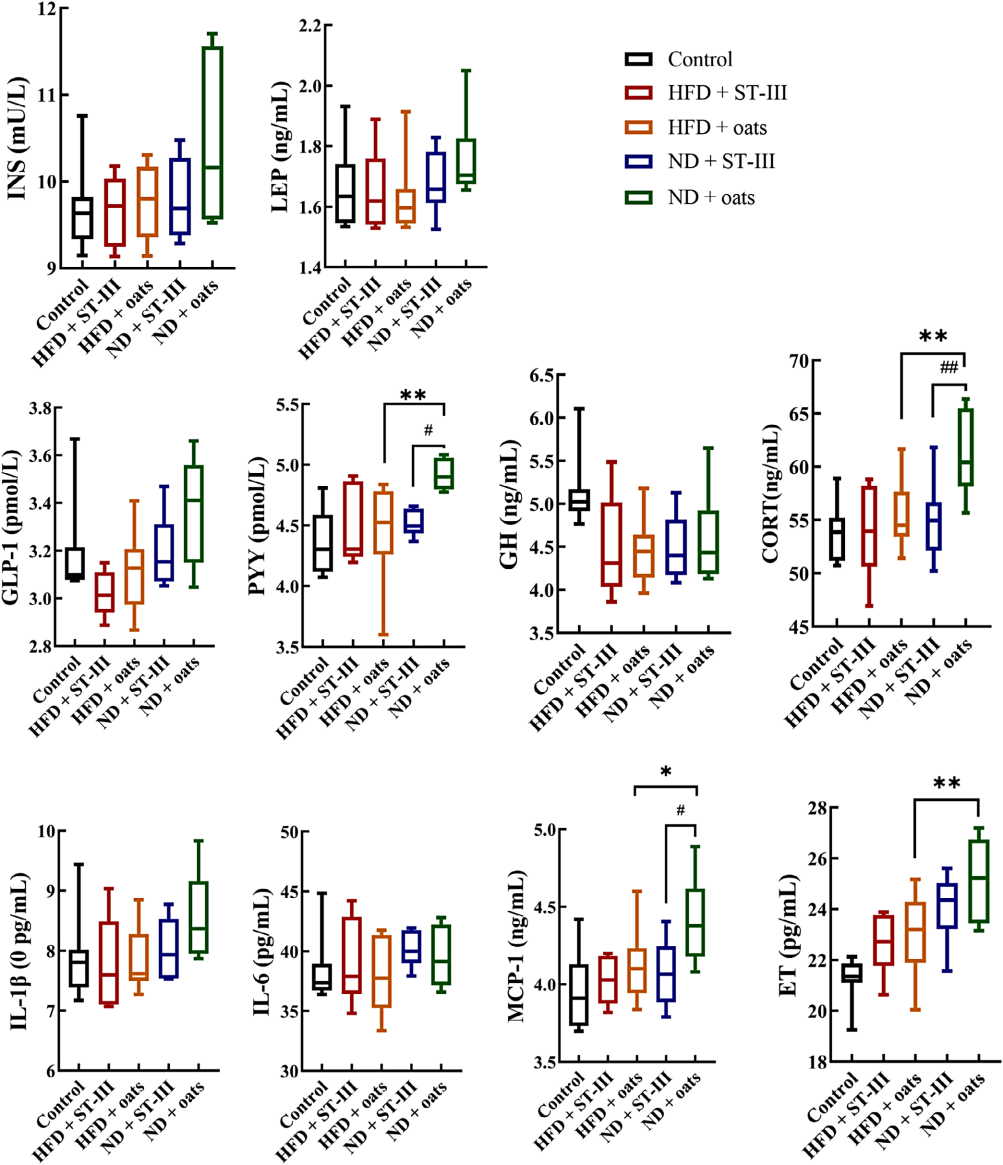
Biochemical analysis (interleukin [IL]‐6, IL‐1β, growth hormone [GH], leptin [LEP], insulin [INS], glucagons‐like peptide 1 [GLP‐1], cortisol, endotoxin [ET], monocyte chemotactic protein 1 [MCP‐1], and peptide YY [PYY]). Each value is expressed as mean ± SD. HFD + ST‐Ⅲ vs. ND + ST‐Ⅲ, HFD + oats vs. ND + oats group, *p < 0.05, **p < 0.01; HFD + ST‐Ⅲ vs. HFD + oats, ND + ST‐Ⅲ vs. ND + oats group, ^*#*^ *p* < 0.05, ^#^^#^ *p* < 0.01. *n* ≥ 6.

This study evaluated inflammatory serum concentrations and compared the levels of inflammatory markers among the groups. As shown in Figure 6, the serum cytokine levels of PYY, CORT, and ET were lower in the HFD + oats group than in the ND + oats group (Figure 6, *p* < 0.05). The study measured 10 cytokines, chemokines, and other inflammatory serum proteins that decreased over the course of the ST‐Ⅲ intervention, including INS, LEP, PYY, GH, CORT, ET, IL‐6, IL‐1β, GLP‐1, MCP‐1, and other inflammatory factors (Figure 6, HFD + ST‐Ⅲ group vs. HFD + oats group, ND + ST‐Ⅲ group vs. ND + oats group). PYY, CORT, and MCP‐1 were significantly decreased in the ST‐Ⅲ intervention group (HFD + ST‐Ⅲ group vs. HFD + Oats group; ND + ST‐Ⅲ group vs. ND + Oats group; *p <* 0.05).

### 3.6 Gut Microbiota Analysis

**FIGURE 7:**
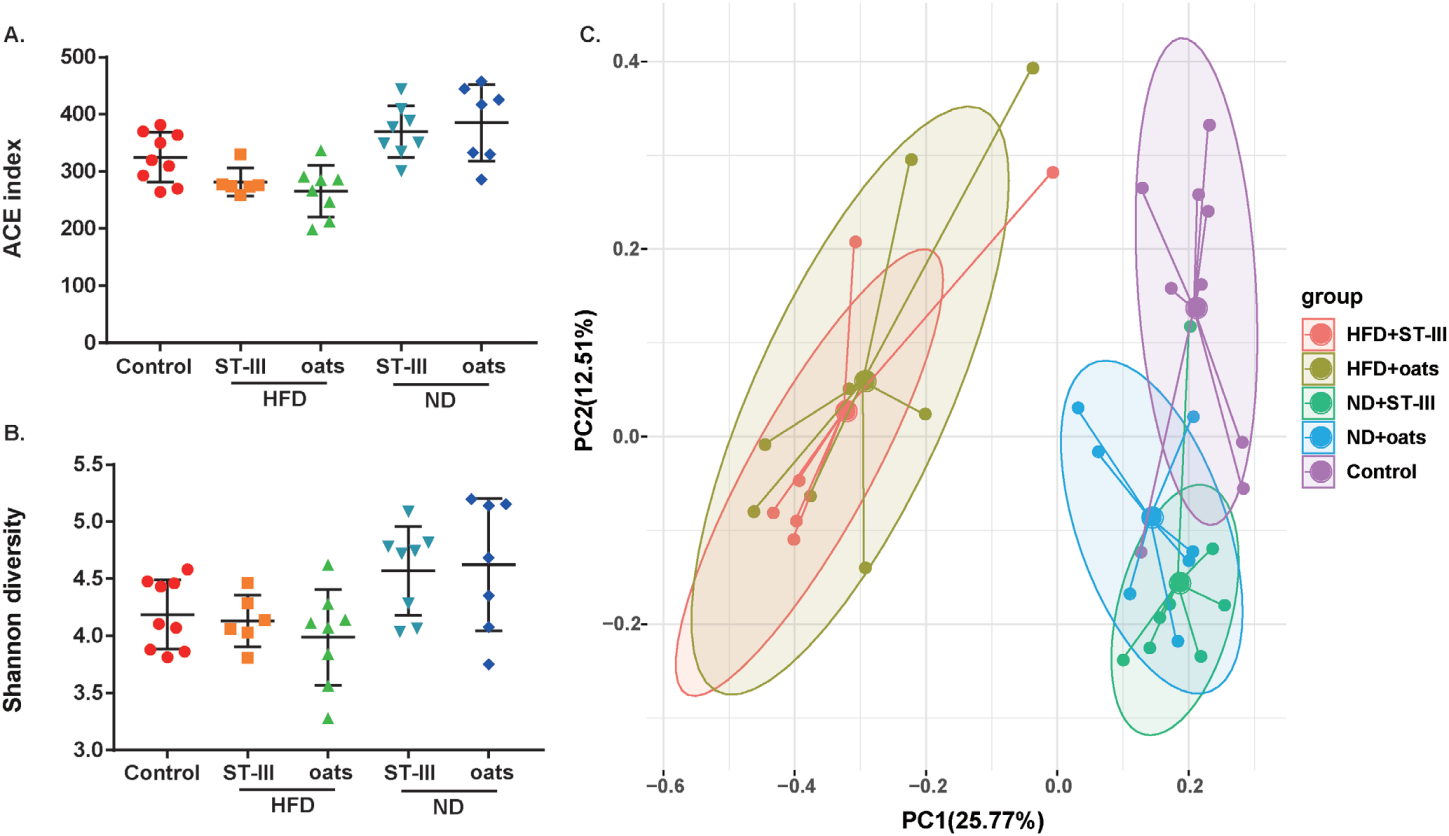
Microbial community comparison among groups. (A) ACE index; (B) Shannon diversity index; (C) principal coordinate analysis (PCoA) of gut microbiota among the five groups based on the Bray–Curtis distance.

A total of 38 samples were analyzed via the V3–V4 region of the 16S rRNA gene amplicon. Finally, 1,109,379 high‐quality reads (18,875 ~ 37,357 reads) were obtained. After the data were normalized, 18,875 16S rRNA genes from each sample were chosen to construct 1828 ASVs (324.4 ± 64.5 ASVs per sample). The richness (ACE index, Figure 7A) of the control samples was significantly different (*p* < 0.05) from that of the other samples, whereas the microbiota diversity was not significantly different (Shannon diversity, Figure 7B). There were no significant differences in richness or diversity between the HFD + ST‐Ⅲ and HFD + oats groups or between the ND + ST‐Ⅲ and ND + oats groups. On the basis of the Bray–Curtis dissimilarity values, there was a significant difference between the control group and the other groups (Figure 7C, *p* = 0.0001).

**FIGURE 8:**
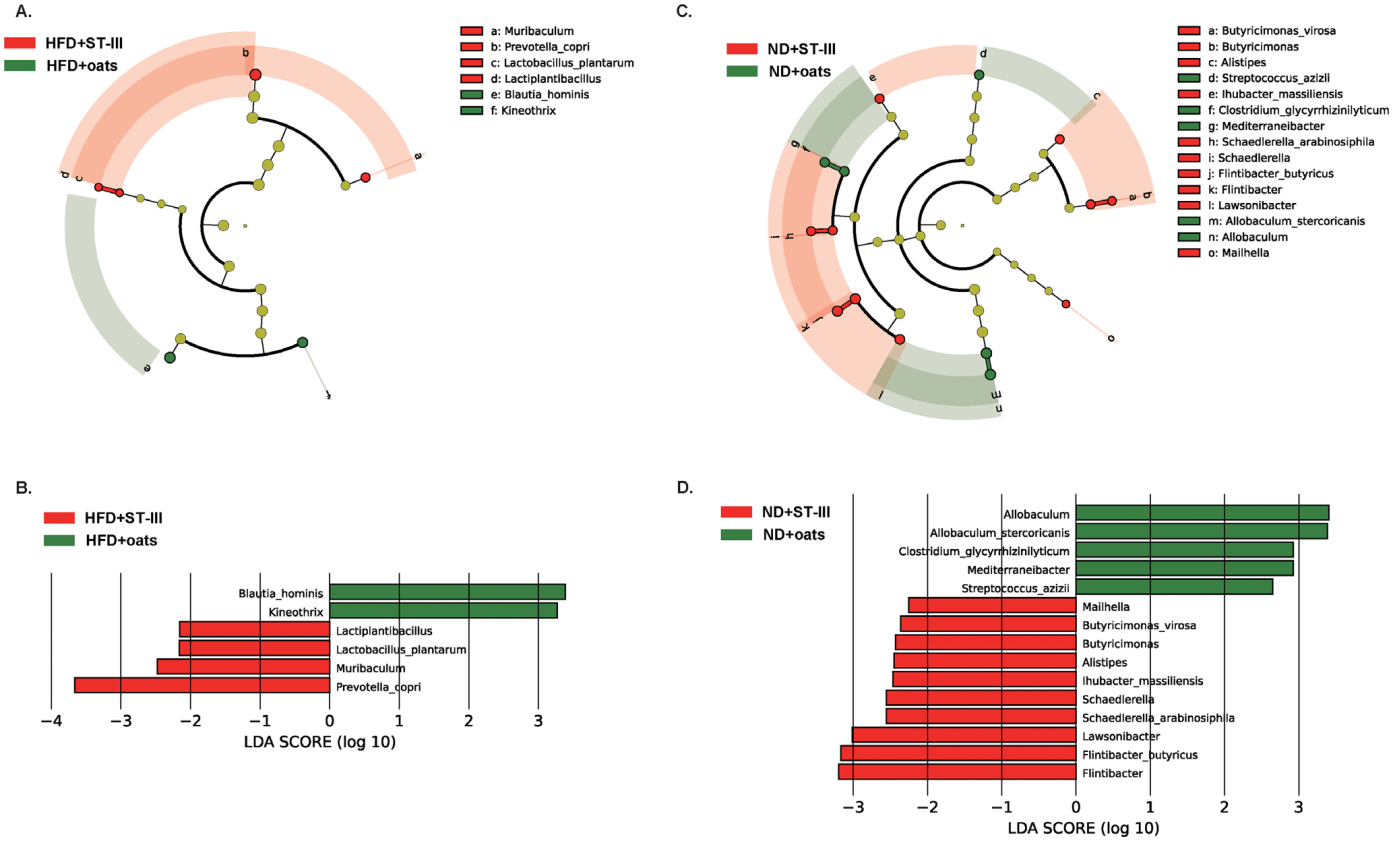
Comparative taxonomic profile between ST‐Ⅲ group and oats group. (A) The cladogram showed differently enriched taxa in HFD + ST‐Ⅲ group and HFD + oats group; (B) LDA scores computed for differentially abundant taxa of HFD + ST‐Ⅲ group and HFD + oats group; (C) the cladogram showed differently enriched taxa in ND + ST‐Ⅲ group and ND + oats group; (D) LDA scores computed for differentially abundant taxa of ND + ST‐Ⅲ group and ND + oats group.

At the genus and species levels, three genera, *Kineothrix*, *Lactiplantibacillus*, and *Muribaculum*; and three species, *Blautia hominis*, *L. plantarum* , and *Prevotella copri* , were significantly different between the HFD + ST‐Ⅲ and HFD + oats groups (Figure 8A,B). Eight genera and seven species were significantly different between the ND + ST‐Ⅲ and ND + Oats groups (Figure 8C,D).

### 3.7 Correlations Between the Microbiota and Biochemical Factors

A total of 30 genera and 37 species were correlated with five biochemical factors (Table S1). In the microbiome compositions, six genera were the most abundant (> 5% at least one group), namely, *Lactobacillus*, *Ligilactobacillus*, *Romboutsia*, *Blautia*, *Turicibacter*, and *Allobaculum*, and only the genus *Blautia* had a positive relationship with blood sugar. A total of 19 species were most abundant in the microbiome (Table 4, > 1% in at least one group), nine of which were correlated with biochemical factors. Four different genera and four different species were correlated with four biochemical factors.

**TABLE 4:** The abundance of species in each group at the end of the study.

Species	Control	HFD + ST‐Ⅲ	HFD + oats	ND + ST‐Ⅲ	ND + oats
	Mean ± SD	Mean ± SD	Mean ± SD	Mean ± SD	Mean ± SD
*Romboutsia timonensis*	0.159506	0.04846799	0.060205298	0.038688742	0.0619943
*Prevotella copri*	0.007935	0.01629139	0.006794702	0.006397351	0.0079546
*Neglecta timonensis*	0.005904	0.00056512	0.001397351	0.010344371	0.0077654
*Lactobacillus reuteri*	0.03811	0.01144371	0.017430464	0.027006623	0.0352015
*Lactobacillus murinus* /*animalis/apodemi*	0.04717	0.01932892	0.021066225	0.103807947	0.0986868
*Lactobacillus johnsonii*	0.037416	0.04116556	0.037834437	0.028993377	0.0365033
*Lactobacillus intestinalis*	0.081742	0.02160706	0.018172185	0.04786755	0.0585355
*Intestinimonas butyriciproducens*	0.012068	0.00140397	0.000834437	0.005033113	0.0104295
*Fusicatenibacter saccharivorans*	0	0.04889183	0.038245033	7.28477E‐05	0.0005449
*Faecalibacterium prausnitzii*	0	0.02571302	0.014337748	0	7.569E‐05
*Escherichia fergusonii*	0.00156	0.03498455	0.000317881	0.001331126	0.0011504
*Enterococcus faecalis*	0.000394	0.01099338	0.00115894	0.000490066	0.0005525
*Clostridium saudiense*	0.02078	0.0050596	0.016900662	0.001940397	0.0008401
*Blautia obeum*	0	0.00513024	0.051112583	0	0.0001135
*Blautia luti*	0	0.02883002	0.022006623	0	0.0010823
*Blautia glucerasea*	0.001419	0.04975717	0.037582781	0.00310596	0.0069555
*Blautia faecis*	0	0.09728918	0.061735099	0.000715232	0.0048515
*Anaerotaenia torta*	0.003079	0.00705519	0.011887417	0.008907285	0.0061079
*Allobaculum stercoricanis*	0.012733	0.08274614	0.102463576	0.00097351	0.0055553

## 4 Discussion

Many studies have reported the anti‐obesity effects of some bacterial strains, such as *L. plantarum* , and their fermentation (Huang et al. 2020, 2022; Martensson et al. 2002). In this study, it was found that feeding a HFD for 9 weeks significantly increased body weight, and the administration of *L. plantarum* ST‐Ⅲ and oats reduced body weight gain and epididymal fat weight (Figures 1 and 3). In previous studies, *L. plantarum* was mostly cultured using MRS medium, which is not directly consumable (Choi et al. 2023; Park et al. 2017). In this study, we used edible pineapple and oats to prepare a medium for culturing *L. plantarum* ST‐Ⅲ, eliminating the need for separation and purification. This allows the cultured product to be directly consumed and potentially applied in future human weight loss studies.

There was no significant difference in body weight between the ST‐Ⅲ and oat groups if they consumed a similar diet (HFD or ND). Importantly, comparing the groups revealed that a HFD promoted a significant increase in the body weight of the rats (*p* < 0.05), indicating that improving dietary structure can achieve weight loss goals. HFD contains more fat and sugar, increasing the intake of substances and energy, altering the balance of gut microbiota, and thereby leading to weight gain (Cani et al. 2007; Turnbaugh et al. 2006). Therefore, weight loss primarily relies on dietary control, and relying solely on *Lactobacillus plantarum* ST‐Ⅲ or oats is insufficient.

In fact, differences in body weight among the groups (the HFD + ST‐Ⅲ group and the ND + ST‐Ⅲ group) became noticeable after ST‐Ⅲ intervention and became significant (*p <* 0.05) after the second week. Differences in body weight among the groups (the HFD + oats group and the ND + oats group) became noticeable after oats intervention and became significant (*p <* 0.05) after the sixth week. Moreover, the weight loss effect of ST‐Ⅲ combined with a ND was much greater than that of oats combined with a ND (Figure 1, HFD + ST‐Ⅲ group vs. ND + ST‐Ⅲ group, HFD + oats group vs. ND + oats group, *p <* 0.05).

Recent studies have shown tha*t L. plantarum* ST‐Ⅲ has cholesterol‐lowering effects in rats (Wang et al. 2012; Huang et al. 2013). Oats can reduce total cholesterol and fat accumulation around the waist in overweight individuals (Ding et al. 2017; Peipei et al. 2010). In this study, the presented results demonstrated that *L. plantarum* ST‐Ⅲ and oats supplementation as a fraction of the total diet are beneficial for preventing weight gain and reducing fat accumulation in HFD‐mediated obesity. However, there was no significant difference in the heart, spleen, kidney, liver, or perirenal fat weight. This is consistent with the hypothesis that HFD‐induced obesity is driven by excessive caloric intake and impaired energy metabolism, which can be mitigated by dietary interventions targeting gut microbiota composition and function (Turnbaugh et al. 2006, Canfora et al., 2019). *L. plantarum* ST‐Ⅲ can modulate lipid metabolism and adipose tissue homeostasis through the production of short‐chain fatty acids (SCFAs), such as acetate, propionate, and butyrate, which are known to enhance energy expenditure and suppress lipogenesis.

Additionally, prolonged HFD consumption is known to increase inflammation both systemically and locally in the liver and intestines (Kyung‐Ah et al. 2012; Phillippi et al. 2022). The serum cytokine levels of GLP‐1, PYY, CORT, and ET were lower in the HFD + oats group (Figure 6, HFD + oats group vs. ND + oats group, *p <* 0.05). This study assessed the levels of PYY, CORT, and MCP‐1, which decreased over the course of the ST‐Ⅲ intervention (Figure 6, HFD + ST‐Ⅲ group vs. HFD + oats group, ND + ST‐Ⅲ group vs. ND + oats group, *p <* 0.05).

This suggests that *L. plantarum* ST‐Ⅲ may exert its anti‐obesity effects through mechanisms beyond simple caloric restriction, such as the modulation of gut hormone secretion (e.g., PYY) and the enhancement of intestinal barrier integrity (Batterham et al. 2002; Cani et al. 2009; Cani et al. 2007). For instance, PYY reduces food intake by acting on hypothalamic appetite centers (Everard et al. 2013).

Considering the known effects of probiotics containing *L. plantarum* on intestinal inflammation, it is plausible that the probiotic treatment in this study functions as an immunomodulator and serves to normalize HFD‐mediated alterations in inflammatory responses.

## 5 Limitations

This research did not establish the obese control group fed with a HFD but without probiotic or oats intervention. Oats were enzymatically hydrolyzed with bromelain. In previous studies, it was found that *L. plantarum* ST‐Ⅲ could grow with pineapple‐added bromelain, but not grow with only oats (results not provided). In the experimental design, the HFD + oats group was intended as the control group, but its weight loss effects were significant. In vitro experiments, the oats could significantly promote the growth of probiotics such as *Bifidobacterium* and *Lactobacillus reuteri* (results not provided). The oral administration of the specially prepared oats could stimulate the growth of native probiotics. So, the oats showed weight loss effects. Future research could focus on native probiotics, combining them with ST‐Ⅲ or enzymatically hydrolyzed oats.

In this study, it is unexpected that the bad health condition is associated with aggressive behaviors such as fighting frequently. Additionally, the rats exhibited extreme fear during the gavage procedure. Thus, the difficulty of long‐term management has produced a high rate of failure for continuing the experiment.

## 6 Conclusion

In conclusion, these data suggest that the *L. plantarum* ST‐Ⅲ used in this study may have beneficial anti‐obesity effects. Therefore, further clinical trials to confirm these effects should be conducted.

## Author Contributions

**Yu Xu:** conceptualization (equal), data curation (equal), visualization (equal), visualization (equal), writing – original draft (equal), writing – original draft (equal), writing – review and editing (equal), writing – review and editing (equal). **Hongfa Zhang:** conceptualization (equal), data curation (equal), validation (equal), writing – original draft (equal), writing – review and editing (equal). **Mingjie Li:** data curation (equal), visualization (equal), writing – review and editing (equal). **Zhaowei Chen:** methodology (equal), writing – review and editing (equal). **Yuezhu Wang:** formal analysis (equal), writing – review and editing (equal). **Rongfu Yang:** methodology (equal). **Sheng Li:** methodology (equal). **Shiyu Ma:** methodology (equal). **Chunping You:** funding acquisition (equal), project administration (equal), supervision (equal), writing – review and editing (equal). **Huajun Zheng:** funding acquisition (equal), project administration (equal), supervision (equal), writing – review and editing (equal).

## Ethics Statement

The experimental protocols involved were all approved by the Shanghai Institute of Planned Parenthood Research Animal Ethics Committee (Protocol Number: 2020‐34) and followed institutional animal care.

## Conflicts of Interest

The authors declare no conflicts of interest.

## Supporting information


Table S1.

## Data Availability

The raw 16S rRNA gene sequencing data have been submitted to the GenBank Sequence Read Archive (accession number PRJNA1132766).
