# Supplementary material for: The Effect of Probiotics on the Improvement of Body Weight and Fat
Source: Food Sci Nutr. 2025 Aug 6;13(8):e70728. doi: 10.1002/fsn3.70728 (PMC12326422; doi:10.1002/fsn3.70728)
Supplement: Supplementary file 1 — Table S1. [file FSN3-13-e70728-s001.docx]

**Table S1. Correlations between microbiota and biochemical factors.**

| Taxa |  | | p-value | spearman | biochcmical factors |
| --- | --- | --- | --- | --- | --- |
| genus | *Streptococcus* | | 0.039 | -.351* | TG(mmol/L) |
| genus | *Flintibacter* | | 0.008 | -.442** | TG(mmol/L) |
| genus | *Lawsonibacter* | | 0.034 | -.354* | TC(mmol/L) |
| genus | *Lawsonibacter* | | 0.021 | -.382* | LDL(mmol/L) |
| genus | *Mailhella* | | 0.014 | -.412* | TG(mmol/L) |
| genus | *Mediterraneibacter* | | 0.021 | .382* | TC(mmol/L) |
| genus | *Butyricicoccus* | | 0.04 | .355* | Glucose(mmol/L) |
| genus | *Monoglobus* | | 0.032 | -.358* | TC(mmol/L) |
| genus | *Limosilactobacillus* | | 0.032 | .362* | TG(mmol/L) |
| genus | *Enteroscipio* | | 0.032 | .363* | TG(mmol/L) |
| genus | *Parabacteroides* | | 0.032 | -.363* | TG(mmol/L) |
| genus | *Rothia* | | 0.034 | .364* | Glucose(mmol/L) |
| genus | *Enterococcus* | | 0.027 | .368* | LDL(mmol/L) |
| genus | *Faecalibacterium* | | 0.026 | .370* | TC(mmol/L) |
| genus | *Fusicatenibacter* | | 0.021 | .384* | TC(mmol/L) |
| genus | *Streptococcus* | | 0.024 | .386* | Glucose (mmol/L) |
| genus | *Enterococcus* | | 0.025 | .397* | HDL(mmol/L) |
| genus | *Blautia* | | 0.017 | .406* | Glucose(mmol/L) |
| genus | *Oscillibacter* | | 0.014 | -.411* | TG(mmol/L) |
| genus | *Gemella* | | 0.015 | .414* | Glucose(mmol/L) |
| genus | *Collinsella* | | 0.018 | -.415* | HDL(mmol/L) |
| genus | *Anaerobutyricum* | | 0.018 | -.416* | HDL(mmol/L) |
| genus | *Amedibacillus* | | 0.013 | -.418* | TG(mmol/L) |
| genus | *Anaeroplasma* | | 0.009 | -.427** | TC(mmol/L) |
| genus | *Prevotellamassilia* | | 0.007 | .443** | TC(mmol/L) |
| genus | *Negativibacillus* | | 0.011 | -.445* | HDL(mmol/L) |
| genus | | *Faecalibacillus* | 0.007 | -.448** | TG(mmol/L) |
| genus | | *Gemella* | 0.005 | .456** | LDL(mmol/L) |
| genus | | *Aerococcus* | 0.004 | -.463** | TC(mmol/L) |
| genus | | *Adlercreutzia* | 0.003 | .486** | TG(mmol/L) |
| genus | | *Jeotgalicoccus* | 0.002 | -.498** | TC(mmol/L) |
| genus | | *Ruminococcus* | 0.002 | -.499** | TG(mmol/L) |
| genus | | *Prevotella* | 0.002 | -.512** | TG(mmol/L) |
| genus | | *Phocaeicola* | 0.001 | -.533** | TG(mmol/L) |
| species | | *Blautia glucerasea* | 0.035 | -.358* | TG(mmol/L) |
| species | | *Parasutterella excrementihominis* | 0.043 | -.361* | HDL(mmol/L) |
| species | | *Lactobacillus reuteri* | 0.032 | .362* | TG(mmol/L) |
| species | | *Rothia nasimurium* | 0.034 | .364* | Glucose(mmol/L) |
| species | | *Clostridium clostridioforme* | 0.028 | -.365* | LDL(mmol/L) |
| species | | *Enterococcus faecalis* | 0.028 | .367* | LDL(mmol/L) |
| species | | *Blautia faecis* | 0.027 | .370* | TC(mmol/L) |
| species | | *Faecalibacterium prausnitzii* | 0.026 | .370* | TC(mmol/L) |
| species | | *Bacteroides dorei* | 0.036 | .371* | HDL(mmol/L) |
| species | | *Bacteroides uniformis* | 0.027 | -.373* | TG(mmol/L) |
| species | | *Clostridium fimetarium* | 0.025 | -.374* | TC(mmol/L) |
| species | | *Corynebacterium lowii* | 0.034 | .376* | HDL(mmol/L) |
| species | | *Desulfotomaculum guttoideum* | 0.024 | .380* | TG(mmol/L) |
| species | | *Clostridium glycyrrhizinilyticum* | 0.021 | .382* | TC(mmol/L) |
| species | | *Flintibacter butyricus* | 0.01 | -.429* | TG(mmol/L) |
| species | | *Prevotella copri* | 0.023 | -.384* | TG(mmol/L) |
| species | | *Streptococcus azizii* | 0.003 | .499** | Glucose(mmol/L) |
| species | | *Lactobacillus johnsonii* | 0.021 | .383* | LDL(mmol/L) |
| species | | *Fusicatenibacter saccharivorans* | 0.021 | .384* | TC(mmol/L) |
| species | | *Holdemania filiformis* | 0.02 | .386* | TC(mmol/L) |
| species | | *Staphylococcus sciuri* | 0.02 | -.390* | TG(mmol/L) |
| species | | *Blautia producta* | 0.013 | .409* | LDL(mmol/L) |
| species | | *Enterococcus faecalis* | 0.019 | .413* | HDL(mmol/L) |
| species | | *Gemella sanguinis* | 0.015 | .414* | Glucose(mmol/L) |
| species | | *Holdemania filiformis* | 0.012 | .414* | LDL(mmol/L) |
| species | | *Eubacterium hallii* | 0.018 | -.416* | HDL(mmol/L) |
| species | | *Blautia glucerasea* | 0.012 | .427* | Glucose(mmol/L) |
| species | | *Faecalimonas umbilicata* | 0.013 | .433* | HDL(mmol/L) |
| species | | *Anaerotignum lactatifermentans* | 0.008 | -.436** | TC(mmol/L) |
| species | | *Lactobacillus johnsonii* | 0.009 | .438** | TG(mmol/L) |
| species | | *Streptococcus pasteurianus* | 0.008 | -.440** | TG(mmol/L) |
| species | | *Negativibacillus massiliensis* | 0.011 | -.445* | HDL(mmol/L) |
| species | | *Asaccharobacter celatus* | 0.007 | .446** | TG(mmol/L) |
| species | | *Gemella sanguinis* | 0.005 | .456** | LDL(mmol/L) |
| species | | *Aerococcus viridans* | 0.004 | -.463** | TC(mmol/L) |
| species | | *Bifidobacterium animalis* | 0.007 | .465** | HDL(mmol/L) |
| species | | *Parvibacter caecicola* | 0.005 | .468** | TG(mmol/L) |
| species | | *Blautia faecicola* | 0.007 | -.469** | HDL(mmol/L) |
| species | | *Lactobacillus johnsonii* | 0.003 | .478** | TC(mmol/L) |
| species | | *Clostridium hylemonae* | 0.005 | -.487** | HDL(mmol/L) |
| species | | *Clostridium clostridioforme* | 0.003 | -.499** | Glucose(mmol/L) |
| species | | *Jeotgalicoccus halophilus* | 0.002 | -.503** | TC(mmol/L) |
| species | | *Blautia luti* | 0.001 | .524** | TC(mmol/L) |
| species | | *Bacteroides sartorii* | 0 | -.601** | TG(mmol/L) |

Note: * indicates *p*<0.05; ** indicates *p*<0.01.
